# Patient Characteristics, Procedural and Safety Outcomes of Bariatric Surgery in England: a Retrospective Cohort Study—2006–2012

**DOI:** 10.1007/s11695-017-2978-x

**Published:** 2017-10-26

**Authors:** Sun Sun, Oleg Borisenko, Tim Spelman, Ahmed R. Ahmed

**Affiliations:** 1 Synergus AB, Kevinge Strand 20, 182 57 Stockholm, Sweden; 2 Health Outcomes and Economic Evaluation Research Group, Center for Healthcare Ethics, Department of Learning, Information, Management and Ethics, Karolinska Institutet, Stockholm, Sweden; 3 Division of Epidemiology and Global Health, Department of Public Health and Clinical Medicine, Umeå University, Umeå, Sweden; 4 Centre for Population Health, Burnet Institute, Melbourne, Australia; 5 School of Public Health and Preventive Medicine, Monash University, Melbourne, Australia; 6 Imperial College London, London, UK

**Keywords:** Morbid obesity, Bariatric surgery, Hospital data, England, Length of stay, Health outcomes

## Abstract

**Background:**

The objective of the study is to analyze procedural and safety outcomes associated with bariatric surgery and describe the characteristics of patients undertaking bariatric procedures in England between April 2006 and March 2012.

**Methods:**

This is a retrospective cohort study of all adult patients in England diagnosed with obesity and undergoing bariatric surgery as a primary procedure in NHS-funded sites between April 2006 and March 2012 using data sourced from the Hospital Episode Statistics dataset. Length of stay (LOS), 30-day readmission, and post-surgery complication were analyzed as primary outcomes. Socio-demographic background, provider type, procedure volume, and comorbidities were all analyzed as potential explanatory variables.

**Results:**

Gastric bypass (GBP, 12,628) was the most utilized procedure, followed by gastric banding (GB, 6872) and sleeve gastrectomy (SG, 3251). The most prevalent comorbidity was type 2 diabetes (23%). Inpatient mortality was low (≤ 0.15%) for all procedure types. LOS and the risks of both post-operative complication and 30-day readmission were significantly lower for GB, relative to those for GBP and SG. Ethnicity, geographical area, surgery type, and volume were all associated with LOS, risk of readmission, and complication. Provider type and deprivation were further associated with LOS while age correlated with readmission only. An increasing comorbidity burden was associated with an increased risk of both readmission and complication.

**Conclusions:**

Gastric bypass was the most frequently reported procedure in England across the observation period. While utilization across all procedure types increased between 2007 and 2010, overall uptake of bariatric surgery in England represents only a small proportion of the eligible population. Readmission and complication rates were lower for gastric banding relative to those for either gastric bypass or sleeve gastrectomy. The observed inpatient mortality rate was low across all procedure types.

## Background

Obesity and overweight are global health problems associated with significant morbidity and mortality [1]. The World Health Organization (WHO) estimates that approximately 25% of women and 22% of men in Europe were obese in 2014 [2]. This represents a 2 and 3% increase from 2010 figures for females and males, respectively [2]. The obesity rate in England has more than doubled within the past 20 years from 12% in 1993 to 25% in 2014 [3]. The current rate of 25% is the second highest in Europe after Hungary (29% in 2014) [4]. The prevalence of *morbid obesity* in particular (defined as a body mass index (BMI) of at least 40 kg/m^2^, or BMI ≥ 35 kg/m^2^ plus at least one obesity-related complication [5]) has also increased markedly from 1.4% of females and 0.2% of males in 1993 to 3.8 and 1.8% of female and males, respectively in 2014 [3]. Obesity increases the risk of both developing and worsening a large range of associated diseases and comorbidities, including type 2 diabetes mellitus (T2DM) [6, 7], cardiovascular disorders [8, 9], and cancers (particularly endometrial, post-menopausal breast, and colorectal) [10].

Management of obesity involves first-line lifestyle modification including diet, physical activity, and behavioral therapy [5, 11]. This may be supplemented with adjunct pharmacotherapy, which currently includes agents such as orlistat and liraglutide in the UK [5, 11]. Sustained weight loss is typically defined as a total 5–10% loss of body weight, sustained for a minimum of 6 months [12]. These conventional treatments are only partially efficacious in achieving sustained weight loss [13]. In contrast, bariatric surgery has been observed to be more effective in reducing weight in both the short- and long-term among morbidly obese patients relative to non-surgical interventions [12–16]. Importantly, in the context of a patient’s overall comorbidity load, bariatric surgery has also been reported to improve obesity-related comorbidity [17]. Recent analyses of the Hospital Episode Statistics (HES) dataset [18] and the National Bariatric Surgery Registry (NBSR) [19] both reported increased utilization of bariatric surgery. HES reported an increase from 72 to 347 procedures in England between 1996 and 2004 [18] while NBSR reported an increase from approximately 500 to 6500 procedures between 2006 and 2013 across the broader UK and Ireland [19]. This increase was most marked within middle-aged women. A 2010 analysis of HES data reported a marked increase in the number of bariatric procedures performed in England from 238 in 2000 to 2543 in 2007 [19]. However, this observation of an increasing trend ceased in 2011 and was replaced with a steady reduction in HES-reported bariatric procedures in HES between 2011 and 2013 (8794, 8024, and 6384 procedures, respectively) [20].

Analysis of NBSR data observed both a low inpatient mortality rate (< 0.1%) and complication rate (< 3%) following the primary procedure. This compares well with best practice international mortality benchmarks (30-day mortality: Sweden 0.05% [21], Italy 0.2% [22], US 0.3% [23]). Bariatric management of obesity is thus generally considered to be safe. Furthermore, the average post-operative stay has been observed to be no more than 3 days; NBSR reported that for 2011–2013 majority of patients stayed 3 days or less in the hospital after operation (GBP and SG 77%; GB 86%), supporting efficient use of inpatient resources [19].

The objective of this study was to analyze procedural and safety outcomes associated with bariatric surgery and describe the characteristics of patients undertaking bariatric procedures in England between April 2006 and March 2012.

## Methods

### Patients

Data were obtained from the HES Admitted Care database. HES collects patient-level data from all admissions, accident and emergency attendances, and outpatient appointments at National Health Service (NHS) hospitals across England, in addition to centers *funded* by the NHS, which can include a subset of independent providers [24]. Each HES record captures clinical, demographic, administrative, and geographical information linked to the individual episode of care. Diagnosis codes used in HES are based on the International Classification of Disease version 10 (ICD-10) [25]. For each admission episode, patients are assigned a primary diagnosis code and up to 19 associated secondary diagnoses. Patients aged 18 years or above who were diagnosed with obesity (ICD-10: E66.0—obesity due to excess calories, E66.1—drug-induced obesity, E66.2—extreme obesity and hypoventilation, E66.8—other obesity, E66.9—obesity unspecified) and underwent bariatric surgery as a primary procedure in NHS sites or NHS-funded sites between April 2006 and March 2012 were included in the analysis. HES is a patient administrative dataset, and a strict statistical disclosure control is applied in accordance with the HES protocol and all data is anonymized [24].

### Surgery Type

Patients were coded to one of four primary procedure groups based on OPCS-4 codes: GBP, SG, GB, and other procedures (including gastroplasty and duodenal switch). The insertion of gastric balloons was not considered. The full list of OPCS-4 and ICD-10 codes for all procedures and diagnoses is summarized in Table 1.

**Table 1:** OPCS-4 and ICD-10 codes used in the analysis

Type of bariatric surgery	OPCS-4 codes
Gastric bypass	G281, G282, G283, G288, G289, G310, G311, G312, G314, G316, G318, G319, G320, G321, G323, G325, G328, G329, G330, G331, G333, G338, G339, G271, G274, G275, G279, G313, G322, G324, G332, G335, G336, G717
Sleeve gastrectomy	G284, G285
Gastric banding	G303
Other	G018, G022, G023, G024, G025, G028, G029, G032, G033, G034, G035, G036, G038, G272, G273, G278, G301, G302, G304, G308, G309, G315, G481, G482, G485, G486, G491, G492, G493, G498, G499, G513, G716
	ICD-10 codes
Procedure complications	T81, T855, T88, Y60–Y70, Y73, Y74, Y81–Y84
Obesity-related comorbidity	
Abnormal glucose tolerance	R73.0
Degenerative joint disease	M15–M19
Depression	F32, F33
Diabetes type 2	E11
Dyslipidemia	E78
Gallstone	K80
Gastroesophageal reflux disease	K21
Hypertension	I11, I15
Infertility	N97
Obstructive sleep apnea	G47.3

### Outcomes

The primary outcomes of this study were length of stay (LOS), 30-day readmission, and surgery complication. LOS was defined as the duration of the episode of care (or “spell”) in days. Readmission was defined as an emergency readmission of a post-operative patient within 30 days of discharge following admission for a bariatric surgical procedure. Complications related to surgery were defined by clinical experts, based on secondary diagnosis codes (ICD-10) as further detailed in Table 1. It mainly includes complications of procedures (T81); mechanical complication of gastrointestinal prosthetic devices, implants, and grafts (T855); other complications of surgical and medical care, not elsewhere classified (T88); misadventures to patients during surgical and medical care (Y60–Y69); anesthesiology devices associated with adverse incidents (Y70); gastroenterology and urology devices associated with adverse incidents (Y73); general hospital and personal-use devices associated with adverse incidents (Y74); general- and plastic-surgery devices associated with adverse incidents (Y81); other and unspecified medical devices associated with adverse incidents (Y82); surgical and other medical procedures as the cause of abnormal reaction of the patient, or of later complication, without mention of misadventure at the time of the procedure (Y83–84). Inpatient mortality was analyzed as a secondary outcome and was defined as a hospital discharge status of death. Weight and body mass index (BMI) were not available from the HES dataset and thus were unable to be analyzed, as either an outcome or explanatory variable. Similarly, data around patient selection and referral for surgery were likewise unavailable from HES.

### Variables and Definitions

Age was divided into five groups: 18–34 years, 35–44 years, 45–54 years, 55–64 years, and 65 years and above. Ethnic group was defined as either “Caucasian” or “non-Caucasian.” Social and economic disadvantage was quantified using the index of multiple deprivation (IMD) as a continuous variable [26]. Patients were then categorized into five groups based on the quintiles of IMD. Geographical area was based on a regional HES code as follows: London, North England (North West, North East, Yorkshire, and the Humber), Central England (West and East midlands), East England, South England (South West and South East), and other. Procedure volume was calculated according to the total number of procedures carried out at each provider during the study period. Procedure volume was categorized into mutually exclusive quartiles and defined as either “very low” (1–399 procedures), “low” (400–848), “medium” (849–1231), or “high” (1231–1537) volume, consistent with the approach adopted by previous studies [27–29]. Provider type was defined as either NHS trust provider or independent provider. Obesity-related comorbidity was defined based on ICD-10 codes detailed as summarized in Table 1. Cumulative comorbidity burden was captured using the Charlson comorbidity index (CCI) [30].

### Statistical Analyses

Categorical variables were summarized using frequency and percentage. Continuous variables were first assessed for skewness using a Shapiro-Wilk test and summarized using mean and standard deviation (SD) or median and inter-quartile range (IQR) as appropriate. Ordinary least squares (OLS) linear regression of the mean was used to model log-transformed LOS (log_10_(*LOS* + 1)), secondary to its significant skew. Logistic regression was used for a model of the binary readmission and complication outcome variables. For each outcome, adjusted regression models were derived using stepwise forward selection of candidate explanatory variables. Overall goodness-of-fit was examined using the adjusted *R* ^2^ for the linear mean regression and an adjusted McFadden pseudo *R* ^2^ for the logistic modelling. The final adjusted model included both the primary explanatory variable and other significant explanatory variables. Utilization data was analyzed for the observation period for which full-year data was available (i.e., 2007–2011). For all analyses, *p* < 0.05 was considered significant. All analyses were undertaken in R version 3.2.2 [31].

## Results

### Patient Characteristics

A total of 26,420 patients were included in the analysis. Gastric bypass (GBP) was the most frequently reported procedure (48%, 12,658), followed by GB (26%, 6872) and SG (12%, 3251) (Table 2). Mean age at surgery was the highest among patients undergoing SG (45.6 (10.4)). Females accounted mostly across all procedure types and were the highest for GB (80%) and lowest for SG (69%). The highest proportion of procedures were performed in North England (27%, 7231 procedures), followed by South England (24%, 6249 procedures), London (23%, 6078 procedures), Central England (18%, 4788 procedures), and East England (6%, 1649 procedures).

**Table 2:** Conditions for Charlson comorbidity index (CCI)

Conditions	ICD-10 codes	Weight*
Acute myocardial infarction	I21, I22, I23, I252, I258	5
Cerebral vascular accident	G450, G451, G452, G454, G458, G459, G46, I60–I69	11
Congestive heart failure	I50	13
Connective tissue disorder	M05, M060, M063, M069, M32, M332, M34, M353	4
Dementia	F00, F01, F02, F03, F051	14
Diabetes	E101, E105, E106, E108, E109, E111, E115, E116, E118, E119, E131, E136, E138, E139, E141, E145, E146, E148, E149	3
Liver disease	K702, K703, K717, K73, K74	8
Peptic ulcer	K25, K26, K27, K28	9
Peripheral vascular disease	I71, I739, I790, R02, Z958, Z959	6
Pulmonary disease	J40–J47, J60–J67	4
Cancer	C00–C76, C81–C97	8
Diabetes complications	E102, E103, E104, E107, E112, E113, E114, E117, E132, E133, E134, E137, E142, E143, E144, E147	1
Paraplegia	G041, G81, G820, G821, G822	1
Renal disease	I12, I13, N01, N03, N052–N056, N072–N074, N18, N19, N25	10
Metastatic cancer	C77, C78, C79, C80 14 3	14
Severe liver disease	K721, K729, K766, K767 18 3	18
HIV	B20, B21, B22, B23, B24, O987 2 6	2

*If any secondary diagnosis fields contain any condition for both cancer and metastatic cancer, additional 8 weights will be deducted from CCI; if CCI < 0, then set CCI is re-coded as 0

Fifty-four percent of patients were associated with a low IMD. Median (IQR) procedure volume (defined as the count of procedures per provider) was the highest for GBP at 898 (565, 1274) and lowest for GB at 821 (388, 1184).

Most procedures reported in HES were performed by NHS trust providers, accounting for 99% of GB, 97% of SG, and 92% of GBP, with the remainder reported by a small subset of NHS-funded independent providers.

The most frequently reported obesity-related comorbidity across all procedures was T2DM, followed by obstructive sleep apnea, dyslipidemia, depression, degenerative joint disease, and gastroesophageal reflux disease. The percentage of patients having no comorbidities was broadly comparable across all procedure types (64% for GB, 62% for GBP, 61% for SG).

### Utilization Trends

**Fig. 1:**
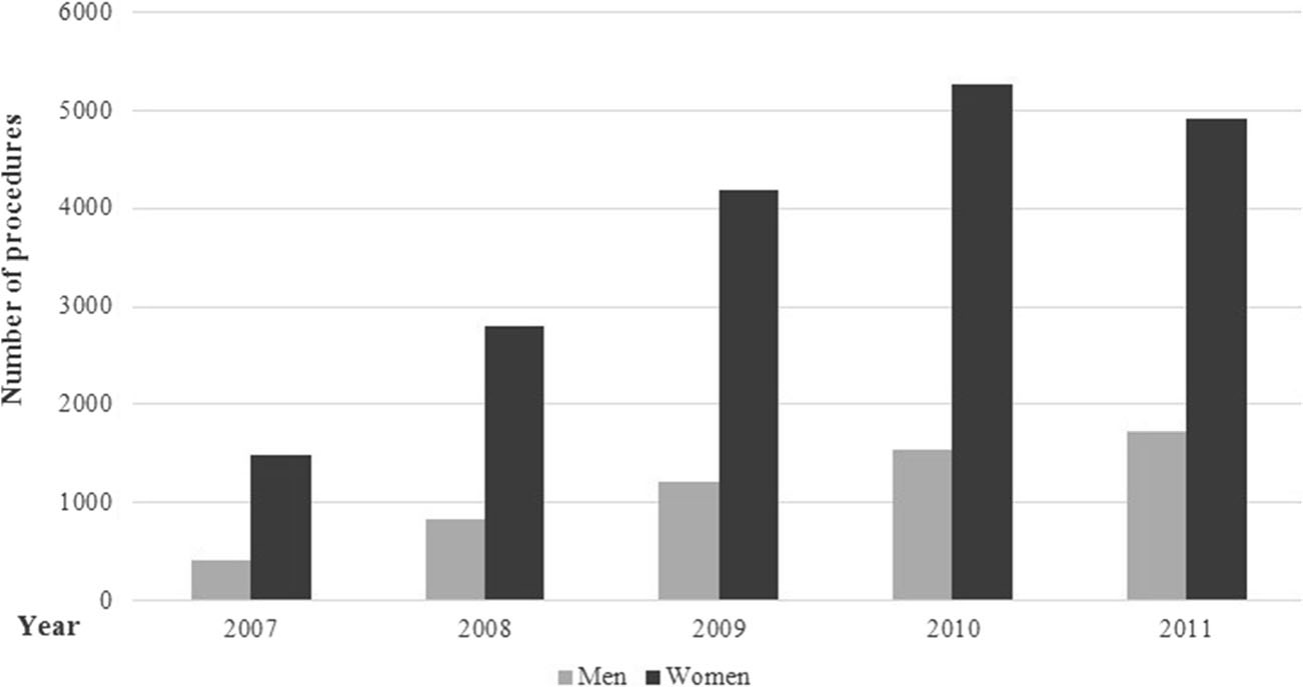
Bariatric surgery utilization trends over time, by sex (year 2006 and 2012 data were excluded from the figure, as the data were not available for the whole year)

**Fig. 2:**
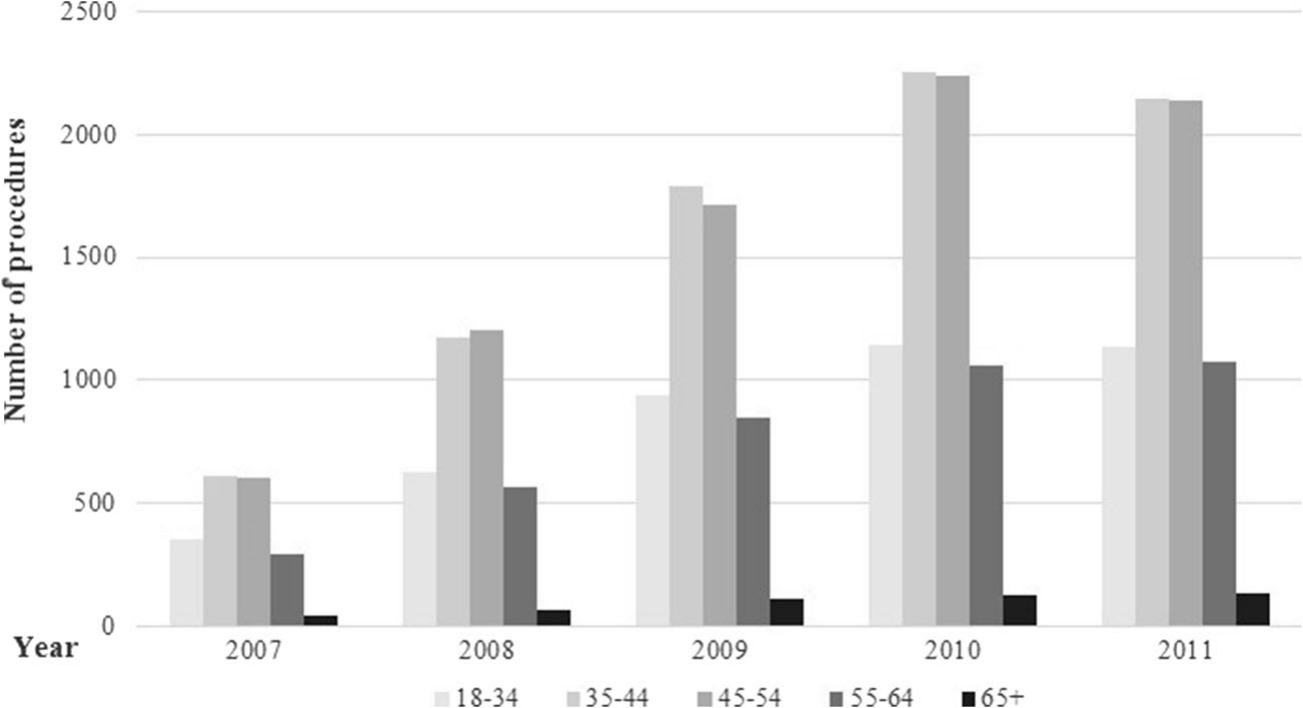
Bariatric surgery utilization trends over time, by age group (year 2006 and 2012 data were excluded from the figure, as the data were not available for the whole year)

**Fig. 3:**
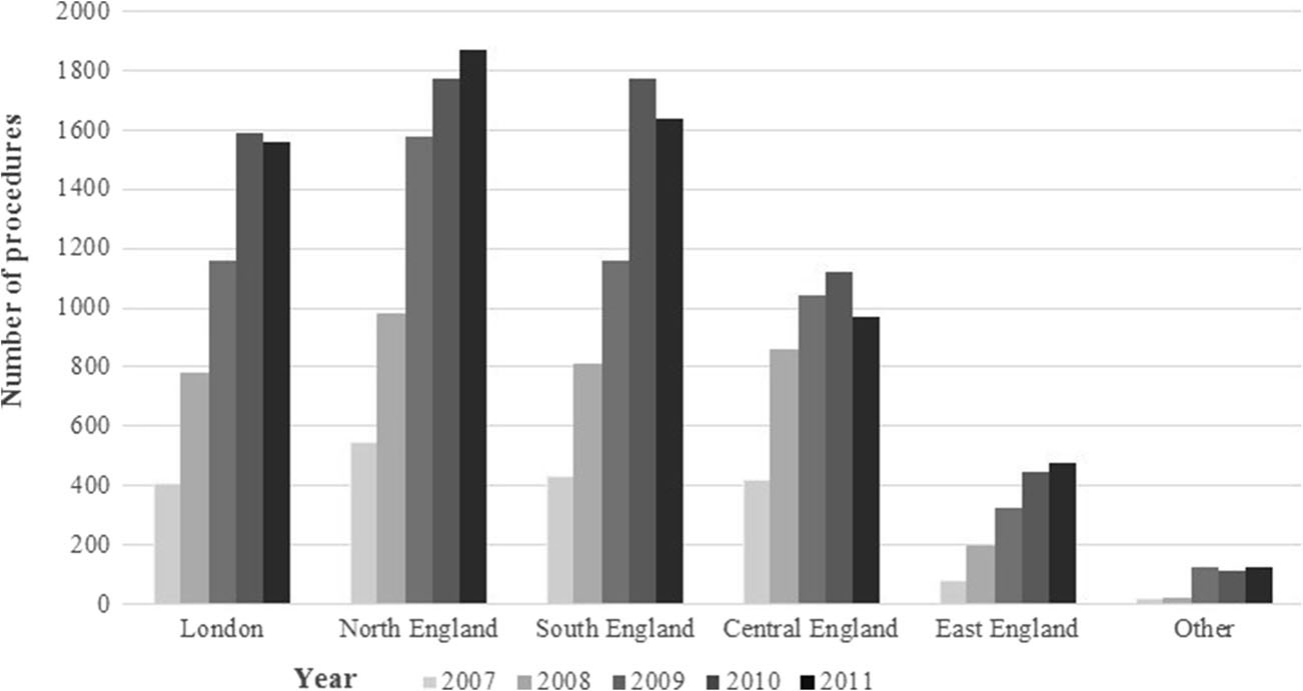
Bariatric surgery utilization trends over time, by geographical area (year 2006 and 2012 data were excluded from the figure, as the data were not available for the whole year)

Utilization trends across 2007 through 2011 by sex, age group, and geographical area are summarized in Figs. 1, 2, and 3, respectively. Females account for a greater proportion of total procedures (18,656 procedures total across a 5-year period compared with 5730 in males) (Fig. 1). The number of bariatric procedures has increased for both sexes across the 2007 to 2010 period; however, a reduction from 2010 to 2011 was observed for females only.

The number of procedures increased steadily over the 2007 to 2010 intervals across all age groups. The rate remained stable between 2010 and 2011 for most age groups except that for patients aged between 45 and 64 years where a reduction was observed (Fig. 2). Patients aged between 35 and 44 years accounted for the highest proportion of procedures (7972 procedures total across a 5-year period). The total number of procedures was the smallest in the 65 years and above age group. Furthermore, the total number of procedures increased across all geographical areas between 2007 and 2010. However, between 2010 and 2011, this increase was only sustained in the North and East England areas, with London and South and Central England all reporting a reduction (Fig. 3). The greatest proportion of procedures were performed in North England (6748 procedures across the 5-year period), followed by South England (5808), London (5498), and Central England (4412), while East England performed the least number of procedures (1522).

### Outcome Analysis

The primary outcomes differed significantly across by procedure type (Table 3). Readmission rates (6% for GB, 10% for GBP and SG) and complication rates (1% for GB, 4% for GBP and SG) were lower for GB relative to either those for GBP or SG. The median (IQR) for LOS was shorter for GB (1 (1,2)), relative to either that for GBP (3 (2,4)) or SG (3 (2,4)). Inpatient mortality was low for all types of procedures (0.15% for GBP, 0.12% for SG, and 0% for GB); however, there were no significant differences across different procedure types. The results for the adjusted (multivariate) regression analyses of the primary LOS, 30-day readmission, and post-surgery complication end points are summarized in Table 4.

**Table 3:** Patient characteristics, by type of surgery

Variables	Surgery type			
	Gastric bypass (*n* = 12,628)	Sleeve gastrectomy (*n* = 3251)	Gastric banding (*n* = 6872)	Other (*n* = 3639)
Individual characteristics				
Age, mean (SD)	44.3 (10.1)	45.6 (10.4)	44.3 (10.7)	44.0 (10.4)
Age group, *n* (%)				
18–34 years	2138 (17)	513 (16)	1247 (18)	676 (19)
35–44 years	4204 (33)	934 (29)	2276 (33)	1203 (33)
45–54 years	4192 (33)	1149 (35)	2083 (30)	1140 (31)
55–64 years	1947 (15)	570 (18)	1068 (16)	555 (15)
65 years and above	177 (1)	85 (3)	198 (3)	65 (2)
Female sex, *n* (%)	9743 (77)	2242 (69)	5478 (80)	2690 (74)
Geographical area, *n* (%)				
North England	4173 (33)	754 (23)	1002 (15)	1302 (36)
Central England	1200 (9)	390 (12)	2188 (32)	1010 (28)
East England	1248 (10)	255 (8)	81 (1)	65 (2)
South England	3251 (26)	495 (15)	2200 (32)	303(8)
London	2640 (21)	1237 (38)	1285 (19)	916 (25)
Other	146 (1)	120 (4)	116 (2)	43 (1)
Caucasian ethnicity, *n* (%)	8759 (69)	2149 (66)	4613 (67)	2726 (75)
Index of multiple deprivation (IMD), *n* (%)				
1st quintile (most deprived)	3874 (31)	1016 (32)	1751 (26)	1317 (37)
2nd	3077 (25)	815 (26)	1567 (23)	925 (26)
3rd	2414 (19)	552 (18)	1345 (20)	610 (17)
4th	1830 (15)	423 (14)	1124 (17)	462 (13)
5th quintile (least deprived)	1311 (10)	322 (10)	966 (14)	281 (8)
Clinical characteristics				
Provider type, *n* (%)				
NHS trust provider	11,659 (92)	3166 (97)	6826 (99)	3517 (97)
Independent provider	999 (8)	85 (3)	46 (1)	122 (3)
Procedure volume, median (IQR)	898 (565,1274)	848 (605, 1131)	821 (388, 1184)	709 (388, 1184)
Procedure volume quartile, *n* (%)				
Very low	3006 (24)	633 (19)	1929 (28)	1210 (33)
Low	2803 (22)	1295 (40)	1574 (23)	945 (26)
Medium	3618 (29)	650 (20)	2589 (38)	703 (19)
High	3227 (26)	670 (21)	778 (11)	778 (21)
Comorbidity, *n* (%)				
T2DM	3004 (24)	780 (24)	1595 (23)	854 (23)
Obstructive sleep apnea	2016 (16)	511 (16)	1038 (15)	584 (16)
Dyslipidemia	1206 (10)	306 (9)	672 (10)	349 (10)
Depression	1061 (8)	295 (9)	581 (8)	295 (8)
Degenerative joint disease	822 (6)	186 (6)	447 (7)	219 (6)
Gastroesophageal reflux disease	603 (5)	169 (5)	329 (5)	178 (5)
Charlson comorbidity index (CCI), *n* (%)				
No comorbidity (0)	7832 (62)	1983 (61)	4428 (64)	2265 (62)
Moderate (1–5)	3846 (30)	949 (29)	1965 (29)	1101 (30)
High (≥ 6)	980 (8)	319 (10)	479 (7)	273 (8)

**Table 4:** Surgery outcomes, by type of surgery complication outcomes

	Surgery type			
	Gastric bypass (*n* = 12,628)	Sleeve gastrectomy (*n* = 3251)	Gastric banding (*n* = 6872)	Other (*n* = 3639)
Inpatient mortality, *n* (%)	19 (0.15)	4 (0.12)	0 (0)	2 (0.05)
Readmission, *n* (%)	1279 (10)	334 (10)	443 (6)	498 (14)
Complications, *n* (%)	494 (4)	123 (4)	76 (1)	114 (3)
Length of stay (days), median (IQR)	3 (2, 4)	3 (2, 4)	1(1, 2)	2 (2, 4)

#### Length of Stay

Medium procedure volume was associated with a 0.04 log unit (0.1 day) decrease in LOS (*β*-coefficient − 0.040; CI − 0.0486, − 0.0322) relative to very low volume (Table 5). High volume was associated with a 0.07 log unit (0.18 day) increase in LOS (*β* 0.072; CI 0.063, 0.081). Independent providers were associated with a 0.06 log unit (0.15 days) decrease in LOS (*β* − 0.062; CI − 0.076, − 0.047), relative to the NHS trust providers. Geographical location, IMD, and ethnicity were also associated with LOS. However, while these marginal differences were statistically significant, they were too small to represent clinical significance. Both GB (*β* − 0.087; CI − 0.094, − 0.080) and SG (*β* − 0.009; CI − 0.019, − 0.0003) were associated with small decreases in LOS relative to GBP, adjusting for all other model covariates; however, these associations were similarly not clinically significant. There was no association between LOS and either age, sex, or CCI.

**Table 5:** Adjusted regression analyses for LOS, 30-day readmission, and post-surgery complication

	Length of stay			30-day readmission		Post-surgery complication	
	*β*-estimate	Exponentiated *β*	95% CI	OR	95% CI	OR	95% CI
Intercept	0.5389	2.4586	(0.5256, 0.5522)	0.1694	(0.1398, 0.2050)	0.0451	(0.0322, 0.0629)
Surgery type^a^							
Sleeve gastrectomy (SG)	− 0.0094	0.0219	(− 0.0185, − 0.0003)	0.9792	(0.8579, 1.1150)	0.9137	(0.7392, 1.1211)
Gastric banding (GB)	− 0.0867	0.2210	(− 0.0939, − 0.0795)	0.5789	(0.5135, 0.6517)	0.2746	(0.2118, 0.3512)
Other	− 0.0098	0.0228	(− 0.0185, − 0.0011)	1.2569	(1.1188, 1.4103)	0.7411	(0.5942, 0.9171)
Individual characteristics							
Age group^b^							
35–44 years	0.0002	0.0005	(− 0.0080, 0.0084)	0.8680	(0.7709, 0.9782)	0.9731	(0.7927, 1.1993)
45–54 years	− 0.0004	0.0009	(− 0.0086, 0.0078)	0.8999	(0.7994, 1.0139)	0.9128	(0.7413, 1.1280)
55–64 years	− 0.0071	0.0165	(− 0.0167, 0.0025)	0.8128	(0.7041, 0.9377)	1.0035	(0.7876, 1.2775)
65+ years	0.0105	0.0245	(− 0.0102, 0.0313)	0.6851	(0.4817, 0.9486)	0.7490	(0.3903, 1.3063)
Female sex^c^	0.0057	0.0132	(− 0.0008, 0.0122)	0.9672	(0.8790, 1.0655)	1.0634	(0.9022, 1.2588)
Non-Caucasian ethnic group^d^	− 0.0109	0.0254	(− 0.0172, − 0.0045)	0.8211	(0.7448, 0.9044)	0.7143	(0.5989, 0.8484)
Geographical area^e^							
North England	0.0299	0.0713	(0.0215, 0.0383)	0.8907	(0.7893, 1.0053)	0.7805	(0.6294, 0.9683)
Central England	− 0.0743	0.1866	(− 0.0837, − 0.0650)	1.1254	(0.9833, 1.2877)	1.1529	(0.9036, 1.4685)
East England	− 0.0154	0.0361	(− 0.0294, − 0.0015)	0.7668	(0.6156, 0.9500)	1.1608	(0.8528, 1.5726)
South England	− 0.0144	0.0337	(− 0.0231, − 0.0056)	0.7689	(0.6719, 0.8796)	1.0543	(0.8366, 1.3280)
Index of multiple deprivation (IMD)^f^							
2nd	0.0031	0.0072	(− 0.0045, 0.0106)	0.9922	(0.8889, 1.1071)	0.9726	(0.8039, 1.1753)
3rd	0.0044	0.0102	(− 0.0039, 0.0128)	0.9038	(0.7977, 1.0228)	0.8932	(0.7192, 1.1052)
4th	0.0158	0.0371	(0.0068, 0.0248)	0.8781	(0.7649, 1.0062)	0.8290	(0.6503, 1.0497)
5th quintile (least deprived)	0.0025	0.0058	(− 0.0077, 0.0127)	0.9257	(0.7918, 1.0790)	0.9005	(0.6884, 1.1672)
Clinical characteristics							
Independent provider^g^	− 0.0616	0.1524	(− 0.0761, − 0.0471)	0.9803	(0.7940, 1.2031)	0.6966	(0.4570, 1.0278)
Procedure volume^h^							
Low	− 0.0080	0.0186	(− 0.0166, 0.0006)	0.9384	(0.8282, 1.0635)	1.0709	(0.8620, 1.3324)
Medium	− 0.0404	0.0975	(− 0.0486, − 0.0322)	0.8992	(0.7962, 1.0158)	0.6176	(0.4883, 0.7802)
High	0.0720	0.1803	(0.0627, 0.0813)	0.7843	(0.6838, 0.8991)	1.0414	(0.8313, 1.3051)
Charlson comorbidity index (CCI)^i^							
Moderate (1–5)	− 0.0046	0.0106	(− 0.0108, 0.0016)	1.0802	(0.9836, 1.1855)	1.4773	(1.2648, 1.7239)
High (≥ 6)	0.0015	0.0035	(− 0.0091, 0.0122)	1.6485	(1.4330, 1.8908)	1.8661	(1.4792, 2.3333)
Adjusted *R* square^j^	0.2777			0.0067		0.0143	

Reference group

^a^Surgery type, gastric bypass (GBP)

^b^Age group, 18–34 years

^c^Sex, men

^d^Ethnic group, Caucasian

^e^Geographical area, London

^f^Index of multiple deprivation, 1st quintile (most deprived)

^g^Provider type, NHS trust

^h^Procedure volume, very low (1–399)

^i^Charlson comorbidity score, no comorbidity (0)

^j^Adjusted *R* ^2^ for model with LOS as outcome, adjusted McFadden pseudo *R* ^2^ for model with 30-day readmission and post-surgery complication as outcomes

#### Thirty-Day Readmission

Only a high procedure volume (OR 0.784; CI 0.684, 0.899) was associated with significantly lower odds of readmission relative to very low volume. A high CCI (OR 1.649; CI 1.433, 1.891) was associated with higher odds of readmission, relative to “no comorbidity”. Non-Caucasians were associated with significantly lower odds of readmission relative to Caucasians (OR 0.821; CI 0.745, 0.904). Relative to London, East (OR 0.767; CI 0.616, 0.950) and South England (OR 0.769; CI 0.672, 0.890) had significantly lower odds of readmission. The odds of readmission in East and Central England did not differ significantly from that in London. All age groups except for age group 45–54 years were inversely correlated with odds of readmission with the oldest patients (aged 65 years or above) associated with the lowest odds (OR 0.685; CI 0.482, 0.949). GB was associated with significantly lower odds of 30-day readmission relative to GBP (OR 0.579; CI 0.514, 0.652), adjusting for all other model covariates. There was no significant difference in risk of readmission between SG and GBP. There was no association between readmission and sex, deprivation, or provider type.

#### Post-surgery Complications

Medium procedure volume was associated with significantly lower odds of post-operative complication relative to very low volume (OR 0.618; CI 0.488, 0.780), while neither low nor high volume differed from very low volume. Both moderate (OR 1.477; CI 1.265, 1.724) and high (OR 1.866; CI 1.479, 2.333) CCI were associated with significantly higher odds of post-surgery complication, relative to no comorbidity. Non-Caucasians were associated with significantly lower odds of post-surgery complication relative to Caucasians (OR 0.714; CI 0.599, 0.848). GB was associated with significantly lower odds of post-surgery complication relative to GBP (OR 0.275; CI 0.212, 0.351), adjusting for all other model covariates. There was no significant difference in the risk of post-surgery complication between SG and GBP. There was no association between risk of post-surgery complication and age, sex, geographical area, provider type, or deprivation.

## Discussion

Based on NICE guidelines, approximately two million people in England would presently qualify for bariatric surgery [32]. However, the actual number of bariatric procedures undertaken nationally each year represents only a small proportion of the eligible population, with the population-standardized bariatric surgical rate in England of 117 per million of the population being comparatively low relative to that in other European settings [33]. Furthermore, the relative distribution of procedure types in England was far more mixed. While GBP was the most frequently reported procedure across the observation period (48%), this was considerably smaller than the proportions reported in Sweden, Denmark, and Belgium where GBP clearly dominates (98, 96, and 80%, respectively) [33]. By contrast, GB was used relatively frequently in England (26%), with only France (19%) and Italy (37%) reporting comparable utilization levels [33]. While overall utilization across all procedure types increased across 2007–2010, the late decline in bariatric procedures between 2010 and 2011 was primarily driven by a reduction in the number of GB procedures undertaken. A recent analysis of the NBSR supports this observation, albeit over a longer time period, reporting an overall decline from 40% in 2007 to 15% in 2013 [19].

Our study observed that LOS and the risk of both post-operative complication and readmission were significantly lower for GB. While this likely reflects the less invasive nature of banding, studies of long-term outcomes up to 15 years post-surgery in GB patients have reported relatively low success rates in terms of sustained weight loss [34]. Of the patient factors assessed, comorbidity level, deprivation index, and ethnicity were all associated with the study outcomes. There was no association between gender and any of the outcomes tested. However, older age at the time of procedure was associated with a reduction in the risk of any readmission. Neither the NICE guidelines nor precedent literature support older age (> 65 years) in itself as a contraindication for surgery [35–37]. However, this diverse range of both patient and provider factors observed in our analysis to associate with post-operative outcome, independent of the procedure type, supports existing guidelines recommending case-by-case assessment of older bariatric surgery candidates [35].

With regard to provider factors, NHS-funded procedures undertaken by independent providers were associated with a significant, albeit marginal, reduction in LOS relative to NHS trust providers on adjusted modelling. LOS varied with geography while the odds of readmission were significantly lower in East and South England relative to London, independent of provider volume. No significant differences in the risk of post-operative complication were found across the different geographical areas. An increasing provider-level procedure volume was also associated with a decreasing risk of readmission. Post-operative complication was also significantly lower for medium procedure volume relative to very low volume providers. This observed link between provider factors and post-operative course supports arguments for expanding minimum standards around infrastructure and volume, in order to optimize post-surgical outcomes of surgery [38, 39]. The International Federation for the Surgery of Obesity and Metabolic Disorders (IFSO) recommends at least 100 surgical cases per year including revisional cases per qualified provider [38]. Similarly, the American Society for Bariatric Surgery (ASBS) requires at least 125 bariatric surgical cases per year per qualified provider, with each surgeon performing a minimum of 50 cases per year and having a total experience of at least 125 cases as the primary surgeon [40, 41]. However, no recommendations regarding minimum number of surgeon and unit volumes are presently available from relevant professional surgical associations such as the British Obesity and Metabolic Surgery (BOMSS) in the UK [42]. In our study, patients treated by high volume providers were associated with longer LOS relative to very low providers. This may in part reflect higher volume providers handling a disproportionately larger proportion of more complicated or riskier cases.

Bariatric surgery is considered a generally safe management option for morbidly obese patients, although health outcomes might vary by surgery type, patient characteristics, study period, and setting [43]. In a meta-analysis of cohort studies and clinical trials from Europe, North America, South America, Australia/New Zealand, and Asia, the total mortality at ≤ 30 days was 0.28% (95% CI, 0.22–0.34) and total mortality at ≥ 30 days to 2 years was 0.35% (95% CI, 0.12–0.58) [43]. In the present study, the observed inpatient mortality rate in England (< 0.2%) was comparable to NBSR-reported rates reported across a similar observation period [44]. While banding may be a preferred option given its relatively low LOS, complication, and mortality rate, it may be less effective than GBP and SG with regard to long-term sustained weight loss and may be associated with a higher rate of revision beyond 7–15 years post-implant [14, 34, 45, 46].

### Limitations

The HES database represents an important and useful source of information, but it does have some limitations. Patient-level BMI and weight data were not available in the HES dataset. Thus, we were unable to study sustained weight loss as an end point, nor adjust the LOS, readmission, or complication models for potential differences in BMI between patient groups undergoing each procedure type. Similarly, long-term complication data and post-banding were not available to analyze trends in revisional surgery nor were data available on the long-term impacts of surgery on T2DM. Non-NHS-funded bariatric surgery activities undertaken by independent providers were not available from HES and thus not included in the analysis. While our observational study employed a retrospective design, the distribution of key patient demographic and clinical characteristics including comorbidities and provider factors were generally well balanced across each of the procedure types considered. There was thus no obvious signal suggesting a marked selection bias secondary to healthier patients being preferentially selected for any one particular type of procedure. Finally, the small number of mortality events prohibited any adjusted analysis secondary to underpowering, while utilization trends were not analyzed post 2011.

In conclusion, gastric bypass was the most frequently reported procedure in England across the observation period. While utilization across all procedure types increased between 2007 and 2010, overall uptake of bariatric surgery in England represents only a small proportion of the eligible population. Readmission and complication rates were lower for gastric banding relative to those for either gastric bypass or sleeve gastrectomy. The observed inpatient mortality rate was low across all procedure types.
